# Enhanced light absorption by mixed source black and brown carbon particles in UK winter

**DOI:** 10.1038/ncomms9435

**Published:** 2015-09-30

**Authors:** Shang Liu, Allison C. Aiken, Kyle Gorkowski, Manvendra K. Dubey, Christopher D. Cappa, Leah R. Williams, Scott C. Herndon, Paola Massoli, Edward C. Fortner, Puneet S. Chhabra, William A. Brooks, Timothy B. Onasch, John T. Jayne, Douglas R. Worsnop, Swarup China, Noopur Sharma, Claudio Mazzoleni, Lu Xu, Nga L. Ng, Dantong Liu, James D. Allan, James D. Lee, Zoë L. Fleming, Claudia Mohr, Peter Zotter, Sönke Szidat, André S. H. Prévôt

**Affiliations:** 1 Earth and Environmental Sciences Division, Los Alamos National Laboratory, Los Alamos, New Mexico 87545, USA; 2 Cooperative Institute for Research in the Environmental Sciences and Department of Chemistry and Biochemistry, University of Colorado, Boulder, Colorado 80309, USA; 3 Department of Civil and Environmental Engineering, Carnegie Mellon University, Pittsburgh, Pennsylvania 15213, USA; 4 Department of Civil and Environmental Engineering, University of California, Davis, California 95616, USA; 5 Aerodyne Research, Inc. Billerica, Massachusetts 01821, USA; 6 Department of Chemical Engineering, University of Texas at Austin, Austin, Texas 78712, USA; 7 Department of Chemistry, Boston College, Boston, Massachusetts 02467, USA; 8 Physics Department and Atmospheric Sciences Program, Michigan Technological University, Houghton, Michigan 49931, USA; 9 School of Chemical and Biomolecular Engineering, Georgia Institute of Technology, Atlanta, Georgia 30332, USA; 10 School of Earth and Atmospheric Sciences, Georgia Institute of Technology, Atlanta, Georgia 30332, USA; 11 School of Earth, Atmospheric and Environmental Science, University of Manchester, Manchester M13 9PL, UK; 12 National Centre for Atmospheric Science, University of Manchester, Manchester M13 9PL, UK; 13 Wolfson Atmospheric Chemistry Laboratory and National Centre for Atmospheric Science, University of York, York YO10 5DD, UK; 14 National Centre for Atmospheric Science, Department of Chemistry, University of Leicester, Leicester LE1 7RH, UK; 15 Department of Atmospheric Sciences, University of Washington, Seattle, Washington 98195, USA; 16 Institute for Meteorology and Climate Research, Karlsruhe Institute of Technology, Eggenstein-Leopoldshafen 76344, Germany; 17 Laboratory of Atmospheric Chemistry, Paul Scherrer Institute, Villigen 5232, Switzerland; 18 Lucerne School of Engineering and Architecture, Bioenergy Research, Lucerne University of Applied Sciences and Arts, Horw 6048, Switzerland; 19 Department of Chemistry and Biochemistry and Oeschger Centre for Climate Change Research, University of Bern, Bern 3012, Switzerland

## Abstract

Black carbon (BC) and light-absorbing organic carbon (brown carbon, BrC) play key roles in warming the atmosphere, but the magnitude of their effects remains highly uncertain. Theoretical modelling and laboratory experiments demonstrate that coatings on BC can enhance BC's light absorption, therefore many climate models simply assume enhanced BC absorption by a factor of ∼1.5. However, recent field observations show negligible absorption enhancement, implying models may overestimate BC's warming. Here we report direct evidence of substantial field-measured BC absorption enhancement, with the magnitude strongly depending on BC coating amount. Increases in BC coating result from a combination of changing sources and photochemical aging processes. When the influence of BrC is accounted for, observationally constrained model calculations of the BC absorption enhancement can be reconciled with the observations. We conclude that the influence of coatings on BC absorption should be treated as a source and regionally specific parameter in climate models.

## Abstract (web summary)

Uncertainties in the absorptive properties of black and brown carbon particles limit our understanding of their warming potential. Following an extensive field campaign, Liu *et al*. report that the magnitude of warming is dependent on particle coatings, which vary due to source and photochemical aging.

## Graphical abstract

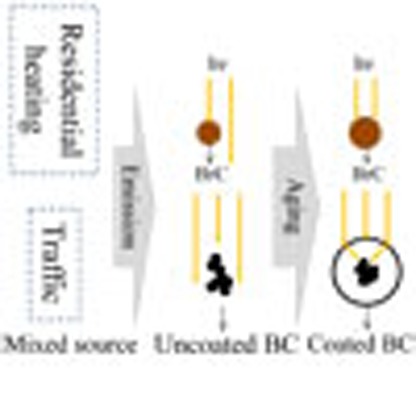

Quantitative prediction of the direct warming effect of absorbing carbonaceous particles is challenging but crucial to assess their climate forcing^1^,^2^,^3^,^4^. The major atmospheric absorbing carbonaceous components are black carbon (BC) and brown carbon (BrC). BC strongly absorbs across the solar spectrum, whereas BrC preferentially absorbs at short wavelengths^2^,^5^. Uncertainties in the absorptive properties of BC and BrC contribute substantially to the total uncertainty estimates of their direct warming effects, in addition to other uncertainties associated with emission estimates, atmospheric transport simulations, and removal rates. Bond *et al*.^6^ suggest that the mass absorption cross-section (*MAC*) of uncoated BC has an uncertainty of 16% at 550 nm. Additional uncertainties in absorption can be introduced as the optical features of BC are continuously modified during atmospheric aging processes. These aging processes mix BC with non-BC species, including BrC. Such mixing, and more specifically formation of coatings on BC particles, can occur over a few hours after BC emission and continue for days^7^. The coatings can enhance the absorption above that of uncoated BC particles through the so-called ‘lensing effect.' The lensing effect has been confirmed by theoretical calculations^8^,^9^,^10^,^11^ and laboratory measurements^12^,^13^,^14^,^15^,^16^ but is not in line with field observations, discussed further below. The inconsistency suggests that using a lensing effect calculated from Mie theory may introduce ∼50% uncertainty in BC's absorption, which can translate to ∼50% uncertainty of modelled BC's direct warming effect. An additional uncertainty of ∼20% may be introduced when including the absorption of BrC^4^, which is ignored by most current climate models. Accordingly, it is important to constrain the dynamic absorptive properties of BC and BrC to improve model predictions.

The influence of BC coatings on BC's absorption has been studied theoretically and experimentally in the laboratory and in the field. Mie theory calculations that assume spherical BC-containing particles with a core–shell configuration, such as those employed by many current climate models, indicate that enhancement factors of up to 3 are plausible^8^,^9^,^10^,^11^. Laboratory studies demonstrate that such absorption enhancement can occur^12^,^13^,^14^,^15^,^16^. Therefore, current climate models typically either calculate an enhancement factor based on simplified mixing-state assumptions^17^,^18^ or assume a constant enhancement value of ∼1.5 for calculating BC absorption^19^,^20^. In contrast, field observations of BC absorption enhancement in urban and coastal California (USA) and Japan^14^,^21^,^22^ yield small values (on average no more than 1.1 at 405, 532, and 781 nm), even when substantial coatings are present^14^,^21^. If true globally, this result questions BC's role as the second most important anthropogenic climate warmer after carbon dioxide^1^,^3^. The difference may be due to a preponderance of ambient BC being only partially encapsulated or attached to the host particle surface^23^. However, larger absorption enhancements of 1.4 (at 532 nm) have been observed in air masses strongly impacted by biomass burning^24^. Because of the paucity of comprehensive ambient measurements, the mechanisms responsible for the model-observation discrepancies remain elusive, underscoring the need for field studies in other representative environments, in particular those with mixed BC and BrC sources.

We perform an in-depth field study of light absorption enhancement factors (*E*_abs_) for BC particles emitted from fossil fuel and residential burning sources in wintertime UK. The measurements are part of the 2012 Clean Air for London (ClearfLo) project^25^ at Detling, a rural site 45 km southeast of central London. We demonstrate substantial light absorption enhancement of BC from our measurements. Through detailed chemical and microphysical analyses, we find that the magnitude of the observed *E*_abs_ strongly depends on the amount of coatings on BC, the BC sources, and the extent of particle aging. We also show that the *E*_abs_ at 405 nm is affected by low-volatility BrC. In addition to the observations, we systematically examine the theoretically calculated *E*_abs_ and determine the refractive indices for BC and BrC that result in agreement between calculated and observed *E*_abs_. Furthermore, single-particle morphological analysis provides mechanistic insight of *E*_abs_ in comparison to previous studies. In light of these analyses, we conclude that the *E*_abs_ is source and regionally dependent. Therefore, parameters used for Mie modelling should be calibrated with observations to account for this effect.

## Results

### Direct observation of *E*_abs_

Observed *E*_abs_ values were calculated using two independent and complementary methods. In one, *E*_abs_ was taken as the ratio between the *MAC* observed for ambient particles and for particles heated at 250 °C. BC is typically considered refractory at these temperatures and the heating is therefore used to remove the part of the coating material that is volatile. Particle heating was achieved by passing particles through a heated thermodenuder (TD). *MAC* is defined as the ratio of the absorption coefficient (*b*_abs_) to refractory BC (rBC) concentration ([rBC]): *MAC*=*b*_abs_/[rBC]. *b*_abs_ at 405 and 781 nm were quantified using photoacoustic spectroscopy^26^ and [rBC] was measured using a single-particle soot photometer (SP2)^27^,^28^. Normalizing *b*_abs_ by [rBC] accounted for particle losses in the TD and temporal variations of *b*_abs_ during each ambient and TD measurement cycle. The observed *E*_abs_ calculated using this method is termed 
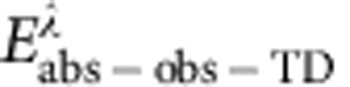
 (λ denotes wavelength). The campaign average 
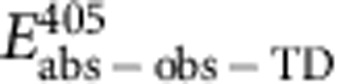
 and 
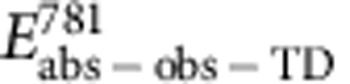
 values were 1.3 and 1.4, respectively, with their time series and frequency distributions shown in Supplementary Fig. 1. The second method was based on the absolute *MAC* values for ambient particles and the estimated enhancement is termed 
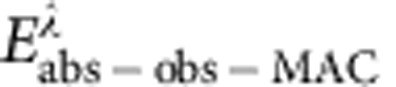
. 
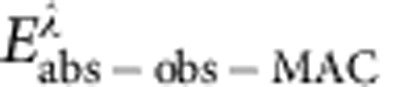
 was calculated as 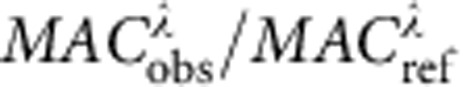, where *MAC*_obs_ is the absolute *MAC* for ambient particles (shown in Supplementary Fig. 2.), and *MAC*_ref_ is the literature reference value for uncoated BC (7.5±1.2 m^2^ g^−1^ at 550 nm)^6^. Using the inverse wavelength dependence relationship for uncoated BC^6^, we derived *MAC*_ref_ values of 10.2±1.6 and 5.3±0.8 m^2^ g^−1^ at 405 and 781 nm, respectively.

Since BrC absorbs at 405 nm but not at 781 nm, the observed *E*_abs_ at 405 nm (using both methods) includes the lensing-driven enhancement and the enhancement induced by semi-volatile BrC, whereas the observed *E*_abs_ at 781 nm represents the lensing-driven enhancement only. A glossary of the abbreviations used in this study is listed in Supplementary Table 1.

### Effects of internal mixing on *E*_abs_

We measured the composition of total non-refractory organic mass (OM) in submicron particles using a high-resolution time-of-flight aerosol mass spectrometer (HR-ToF-AMS)^29^,^30^ and the non-refractory OM internally mixed with BC with a soot particle aerosol mass spectrometer (SP-AMS)^31^. The SP-AMS was operated in laser-only mode, which allows for detection of only rBC-containing particles (Methods). The ratio of non-rBC mass to rBC mass in rBC-containing particles is termed *R*_BC_. Assuming a core–shell particle configuration, *R*_BC_ is a measure of relative coating thickness.

**Figure 1:**
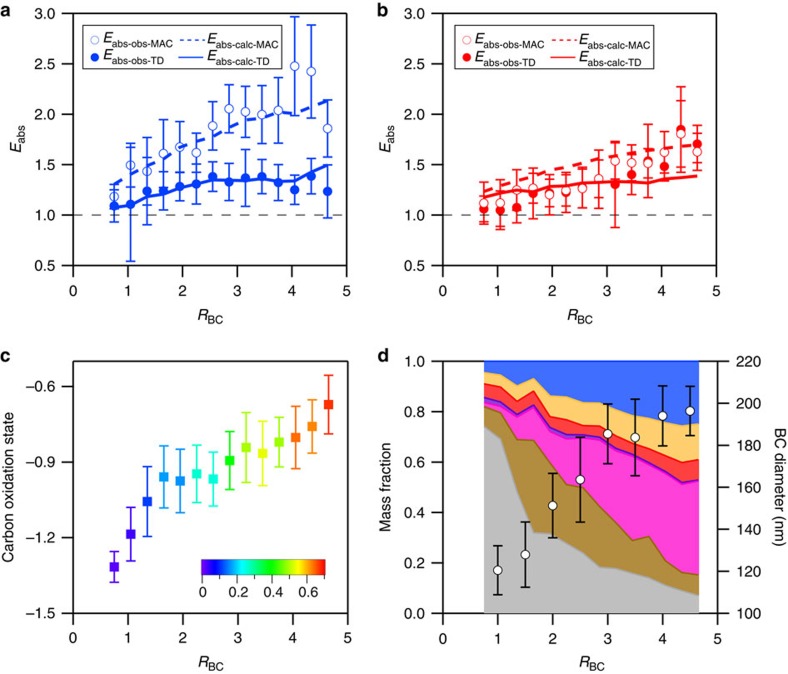
Variation of *E*_abs_ and particle composition as a function of *R*_BC_. (**a**,**b**) show measured and calculated absorption enhancement values versus *R*_BC_ at 405 and 781 nm, respectively. *k*_BrC_ of 0.004 and 0.032 were used for modelling absorption of ambient and thermodenuded organic mass at 405 nm, respectively. (**c**) Particle average oxidation state versus *R*_BC_. The points are coloured by the organic mass fraction of the oxygenated organic aerosol factor, which was derived from factor analysis of the BC-associated OM. (**d**) Mass fraction of the non-refractory components internally mixed with BC (shaded areas) and BC core median volume-weighted diameter (open circles) as a function of *R*_BC_. The colours represent nitrate (blue), ammonium (orange), sulfate (red), chloride (purple), oxygenated organic aerosol factor (pink), solid fuel organic aerosol factor (brown) and hydrocarbon-like organic aerosol factor (grey). The error bars in (**a**–**d**) represent s.d. of the values for each *R*_BC_ interval.

The observed *E*_abs_ at Detling increased substantially with increasing *R*_BC_ (Fig. 1a,b) unlike the previous studies in California^14^,^21^, even though the maximum *R*_BC_ values observed here were three times smaller. In our study, there was good agreement between 
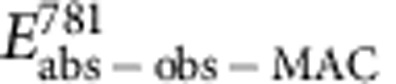
 and 
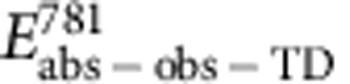
, where BrC is thought to absorb negligibly. This result indicates that a lensing-driven absorption enhancement was, at times, substantial in Detling. It also suggests that residual non-rBC materials did not have a notable impact on absorption by the thermodenuded particles. In contrast, 
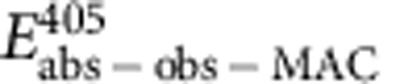
 was notably higher than 
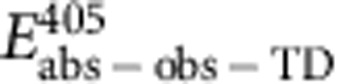
. Furthermore, 
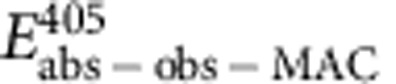
was larger than 
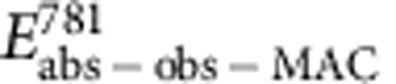
 at a given *R*_BC_. These features resulted from the direct contribution of BrC on the absorption at 405 nm.

As BrC contributes to the absorption at 405 nm, it is necessary to separate the absorption by BrC and evaluate the effect of BrC on *E*_abs_ at 405 nm. *b*_abs_ at 405 nm 
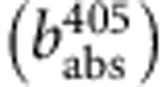
 was apportioned into absorption by residual BC (BC and low-volatility coatings on BC that remain on the particles after thermodenuding at 250 °C), low-volatility BrC that did not fully evaporate in the TD (BrC_LV_), semi-volatile BrC that evaporated in the TD (BrC_SV_), and the increment of BC absorption due to the lensing effect, with their respective fractions of 53, 24, 7 and 16% (Methods). The apportioned values were similar to the apportionment results of Lack *et al*.^24^ at 404 nm inside a wildfire plume (Supplementary Table 2), even though we sampled a mixture of regional fossil fuel and residential burning sources with much lower absorption and OM concentrations. Given that the absorption of BrC_LV_ was 3.4 times the absorption of BrC_SV_ and that the mass of BrC_LV_ was only 9.3% of the mass of BrC_SV_ (measured by the HR-ToF-AMS assuming all OM is BrC), we estimated that the *MAC* of the BrC_LV_ was ∼36 times the *MAC* of BrC_SV_. The much higher *MAC* of BrC_LV_ was likely because BrC_LV_ contained larger molecules with more conjugated carbon bonds (compared with BrC_SV_) that have lower volatility. This result is consistent with a recent study that shows BrC absorption from biomass burning is mostly associated with compounds of extremely low volatility^32^. Since the total non-refractory OM was substantially greater than the non-refractory OM that was associated with rBC, it is likely that much of the BrC was externally mixed with rBC.

The observed *E*_abs_ at 405 nm using the TD method was affected by BrC_LV_. The increase of BrC_LV_ with *R*_BC_ (Supplementary Fig. 3a) suggests a larger contribution of BrC_LV_ to the absorption of thermodenuded particles as the rBC coating thickness increased, resulting in lower 
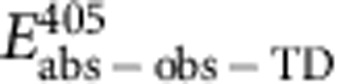
 at high *R*_BC_. As a result, 
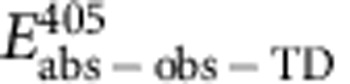
 leveled off for *R*_BC_ values greater than ∼3 (Fig. 1a). In contrast, 
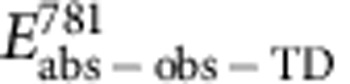
 continuously increased with *R*_BC_ (Fig. 1b) as the absorption of BrC was negligible at 781 nm.

### Dependence of *E*_abs_ on sources and aging

To elucidate the mechanisms causing the enhanced BC absorption and formation of BrC_LV_, the BC-associated OM (measured by the SP-AMS) and total OM (measured by the HR-ToF-AMS) were apportioned into linearly independent components using positive matrix factorization^33^,^34^. The analysis resulted in three factors for both BC-associated OM and total OM, namely oxygenated organic aerosol, solid fuel organic aerosol, and hydrocarbon-like organic aerosol. Based on the mass spectra, correlations with source tracers, and correlations with air mass sources from back trajectories, the oxygenated organic aerosol components were associated with long-range transport and aging of particles, the solid fuel organic aerosol components were associated with solid fuel burning for residential heating including biomass and coal, and the hydrocarbon-like organic aerosol components were associated with traffic emissions. The organic carbon in the total oxygenated organic aerosol component (oxygenated organic carbon) was further separated into fossil and non-fossil fractions using a combination of radiocarbon (^14^C), factor analysis, and BC source analyses (Methods). The apportionment showed that total oxygenated organic carbon (and likely BC-associated oxygenated organic carbon) was largely (73–90%) derived from the non-fossil sources. In addition, we find that the absorption by BrC_LV_ correlated with the total oxygenated organic aerosol component (Supplementary Fig. 3b), indicating that BrC_LV_ may be secondary, as previous laboratory studies proposed^35^,^36^. In contrast, such correlation was not found for BrC_SV_.

The variation of *E*_abs_ with *R*_BC_ was associated with changing sources as well as photochemical processing (Fig. 1c,d). The overall composition of rBC-containing particles varied with *R*_BC_ (Fig. 1d). The relative abundance of secondary components (inorganics and oxygenated organic aerosol component) increased with *R*_BC_, while the relative abundance of primary components (hydrocarbon-like organic aerosol and solid fuel organic aerosol components) decreased. At lower *R*_BC_, the BC-associated OM was dominated by traffic sources and the *E*_abs_ value (∼1.1) was similar to the value observed at much higher *R*_BC_ by Cappa *et al*.^14^, in which fossil fuel combustion is the dominant source for BC. Since the total oxygenated organic carbon was largely from non-fossil sources, the increase in *R*_BC_ was correlated with changing sources that could produce BC particles in distinct size regimes. Given that BC particles from biomass burning are typically larger than those from fossil fuel combustion^37^, the correlation of BC core median volume-weighted diameter (measured by the SP2) with *R*_BC_ (Fig. 1d) was consistent with the oxygenated organic aerosol components being increasingly produced by biomass combustion related sources as the oxygenated organic aerosol concentration increased. The increase of BC diameter with *R*_BC_ may also indicate that coagulation during transport increased the BC size. However, since coagulation rates scale with the square of particle number (which decreases away from sources), and only coagulation between two BC-containing particles can lead to an increase in the BC core size, this hypothesis seems unlikely.

The increase in *R*_BC_ was correlated with an increasing average carbon oxidation state of BC-containing particles (Fig. 1c), which was calculated from the SP-AMS measured atomic O:C and H:C ratios (oxidation state=2 × O:C—H:C)^38^. The atomic O:C and H:C ratios were calculated using the parameterization in Canagaratna *et al*.^39^ Particle oxidation state increases with atmospheric oxidation and was used as a proxy for particle photochemical age here (we do not have the necessary measurements to calculate photochemical age). The correlation of *R*_BC_ with oxidation state suggests that photochemical processes over long timescales were likely important for producing both organic and inorganic coatings on BC.

### Comparison of calculated and observed *E*_abs_

**Figure 2:**
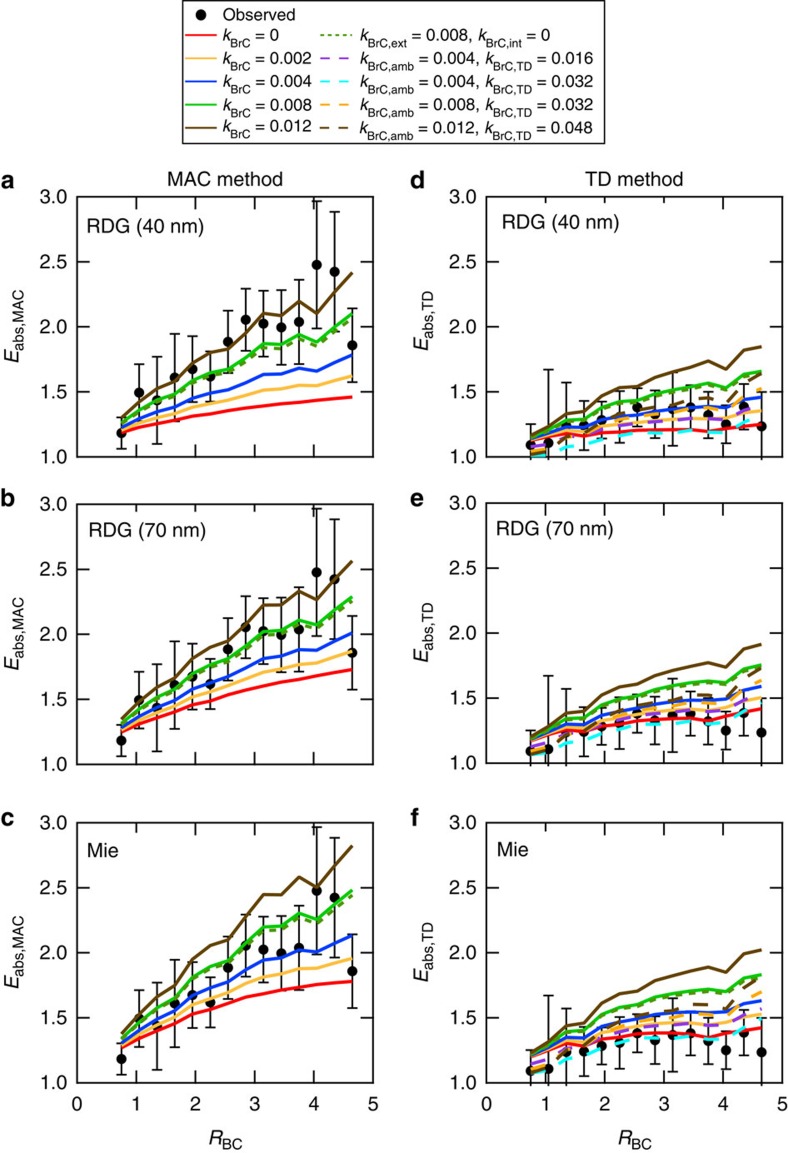
Comparison of observed and modelled *E*_abs,MAC_ and *E*_abs,TD_ at 405 nm. Mie and RDG calculations (assuming BC spherule sizes of 40 and 70 nm) were performed using a range of *k*_BrC_ values that are shown in the legend. *k*_BrC_ indicates the cases in which a single *k*_BrC_ value was assumed for OM; *k*_BrC,ext_ and *k*_BrC,int_ represent the *k*_BrC_ values for OM that was externally and internally mixed with BC, respectively; *k*_BrC,amb_ and *k*_BrC,TD_ represent the *k*_BrC_ values for ambient and thermodenuded OM, respectively. The error bars in (**a**–**f**) represent s.d. of the measured absorption enhancement values for each *R*_BC_ interval.

An important consideration is the extent to which the observations are consistent with Mie theory calculations of BC absorption, as core–shell Mie theory is used in many current climate models. The *E*_abs_ values were calculated using observationally constrained core–shell Mie theory (Methods) by either assuming that no (*E*_abs-calc-MAC_) or some (*E*_abs-calc-TD_) residual material remained both on the BC particles and as externally mixed particles after heating^21^. The *E*_abs-calc-MAC_ and *E*_abs-calc-TD_ were conceptually comparable to *E*_abs-obs-MAC_ and *E*_abs-obs-TD_, respectively, assuming core–shell configuration for both ambient and thermodenuded BC-containing particles. An additional set of calculations were performed under the Rayleigh–Debye–Gans (RDG) approximation: The absorption by both uncoated and coated BC was determined by the properties of the individual spherules that made up a single BC particle^40^. The total absorption by a single particle is approximated as the sum of the absorption from individual spherules comprising a single particle, as calculated from spherical-particle Mie theory. As the size of the spherule was not explicitly known, and further might vary with the BC source, calculations were performed assuming spherules of 40 and 70 nm (diameter) to assess the sensitivity of the results to the assumed spherule size. To account for BrC contributions at 405 nm, calculations were performed using a range of imaginary refractive indices for the OM to find solutions that match the observed values. The base case assumed that OM internally mixed with BC had the same refractive index as that externally mixed from BC, and that the refractive index of the ambient OM was the same as the residual OM after heating. Alternative cases were also considered, and results of the calculations are shown in Fig. 2.

Recalling that *E*_abs-obs-TD_∼*E*_abs-obs-MAC_ at 781 nm, the calculated *E*_abs_ generally reproduced the observed trend with *R*_BC_ (Fig. 1b). Comparison with the observed values at 781 nm showed that: (1) *E*_abs-calc-MAC_ overestimated observations by 14% when *R*_BC_ was smaller than 2.9; (2) *E*_abs-calc-TD_ overestimated observations by 14% for *R*_BC_<1.4 and underestimated observations by 17% for *R*_BC_>3.8; and (3) *E*_abs-calc-TD_ was less dependent on *R*_BC_ compared to *E*_abs-obs-TD_. However, the differences between calculated and observed absorption enhancement were comparable to the measurement uncertainty (15%) of *E*_abs-obs-TD_. The difference between modelled *E*_abs-calc-MAC_ and *E*_abs-calc-TD_ at 781 nm resulted from (theoretical) contributions of residual coatings to the absorption by thermodenuded particles. As the observations suggested negligible contributions of residual coatings, this indicates that the *E*_abs-calc-MAC_ overestimated the absorption enhancement due to the lensing effect for thermodenuded particles at a given *R*_BC_ compared with the observations.

The *E*_abs-calc-MAC_ at 405 nm were lower than the observations when OM was assumed to be non-absorbing for both the Mie and RDG cases (Fig. 2), suggesting a role for BrC absorption. Direct absorption by the internally mixed BrC was very small compared with externally mixed BrC, which was primarily a reflection of the much larger mass concentration of externally mixed OM compared with BC-associated OM. This was assessed by comparing two cases, one with a bulk, campaign average imaginary refractive index (*k*_BrC_) of 0.008 and the other with *k*_BrC_=0 for the internally mixed OM while leaving *k*_BrC_=0.008 for the externally mixed OM. The difference in observed and calculated *E*_abs-MAC_ can be reconciled if the ambient OM was assumed to be slightly absorbing, with a bulk, campaign average *k*_BrC_=0.004 for the Mie case, *k*_BrC_∼0.008 for RDG(70 nm) case, and *k*_BrC_∼0.012 for the RDG(40 nm) case at 405 nm. The larger *k*_BrC_ required for RDG (40 nm) and RDG (70 nm) compared with Mie resulted from the larger initial difference between the *E*_abs-MAC_ values for RDG (40 nm) and RDG (70 nm) when *k*_BrC_=0.

The *E*_abs-TD_ at 405 nm was well-simulated when the OM was assumed non-absorbing, which is inconsistent with the above finding that the OM needed to be absorbing at 405 nm to match the *E*_abs-MAC_ measurements. Alternatively, reasonable quantitative agreement between the observed and calculated *E*_abs-TD_ at 405 nm was obtained when the small amount of residual, low-volatility OM after thermodenuding was assumed to be more absorbing than the ambient OM. Specifically, good agreement (on average within 5%) was obtained for the Mie calculation when *k*_BrC_ of thermodenuded OM (*k*_BrC,TD_) equals 0.032 at 405 nm, while *k*_BrC_=0.004. The *MAC* calculated for the BrC scales approximately linearly with *k*_BrC_, and thus these results indicate the residual OM was approximately 8 times as absorbing per mass as the ambient OM. This finding indicates that the OM components that evaporated were comparably much less absorbing, with an estimated imaginary refractive index of 0.001 at 405 nm assuming volume mixing and that the average residual OM was 8.5% of the total (measured by the HR-ToF-AMS). This result gives a ratio between the *k*_BrC,TD_ and the *k*_BrC_ for the evaporated components of ∼32, consistent with the results from the apportionment of 
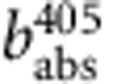
 above. For the RDG calculation, good observation-model agreement can be achieved when the residual OM was six times as absorbing as the ambient OM, that is, *k*_BrC,TD_=0.048 for RDG(70 nm) and *k*_BrC,TD_=0.072 for RDG(40 nm). The overall results indicate that there was a non-negligible lensing-driven absorption enhancement of BC in Detling, and BrC contributed substantially to the observed particle absorption, and thus as well to the observed *E*_abs_ at 405 nm.

### Mechanistic insight of *E*_abs_ from particle morphology

**Figure 3:**
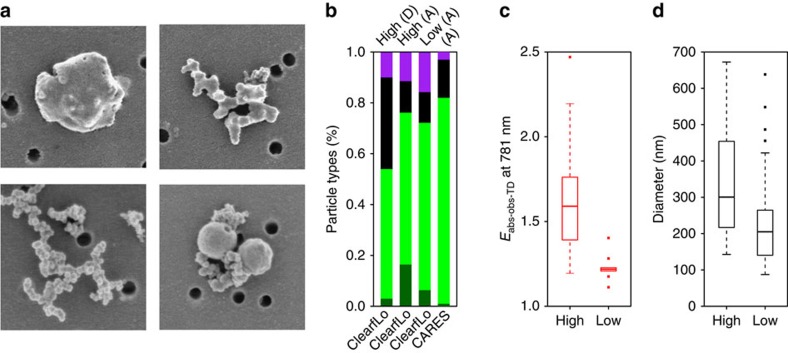
Morphology and statistics of BC-containing particles. (**a**) Representative electron microscopy images of BC-containing particles collected at the Detling site for embedded (top left), partly coated (top right), thinly coated (bottom left) and partially encapsulated and/or surface attached (bottom right) BC particle types. The size of each panel is 1 μm by 1 μm. (**b**) Average number fraction of particle types with the colours indicating embedded (dark green), partly coated (green), thinly coated (black) and partially encapsulated and/or surface attached (purple) BC particle types for the high- and low-*E*_abs-obs-TD_ samples at Detling during ClearfLo and at Sacramento during CARES. The ‘D' and ‘A' in the parenthesis indicate thermodenuded and ambient samples, respectively. (**c**) Box plot of 
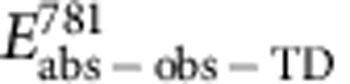
 for the high- and low-*E*_abs-obs-TD_ single particle sampling periods in Detling. (**d**) Box plot of area equivalent diameter of the embedded BC particles for the high-*E*_abs-obs-TD_ samples (#1 and #2 in Supplementary Table 3) and low-*E*_abs-obs-TD_ samples (#3 and #4 in Supplementary Table 3) for the Detling measurements. In the box plots (**c**,**d**), the bounds of each box represent quartiles, and the whiskers extend to the most extreme data points that are within 1.5 interquartile range of the box. The horizontal bars inside the boxes represent the median values.

To develop a mechanistic understanding of the notable dependence of *E*_abs_ on *R*_BC_ at Detling, single-particle images from scanning electron microscopy were analysed. Individual BC particles were classified visually based on their coating and morphologies as ‘embedded', ‘partly coated', ‘thinly coated', and ‘partially encapsulated and/or surface attached' (Fig. 3a), similarly to China *et al*.^41^,^42^ The number fraction of each type was determined (Supplementary Table 3) and the averages are shown in Fig. 3b. (The electron microscopy analysis may exhibit some composition-dependent biases due to possible losses of semi-volatile materials; Methods.)

Two samples were selected for analysis when the *E*_abs-obs-TD_ at 781 nm was high and another two samples were selected during low-*E*_abs-obs-TD_ periods. The 
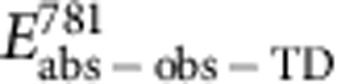
 values for the high- and low-*E*_abs-obs-TD_ samples were 1.67±0.42 (s.d.) and 1.23±0.07 (s.d.), respectively (Fig. 3c). The embedded, partly coated, thinly coated and partially encapsulated and/or surface attached particle types had number fractions of 17%, 60%, 12% and 11% (out of 2187 particles) for the high-*E*_abs-obs-TD_ and 6%, 66%, 12% and 16% (out of 2017 particles) for the low-*E*_abs-obs-TD_ samples. Therefore, the largest difference between the high- and low-*E*_abs-obs-TD_ samples was that the high-*E*_abs-obs-TD_ samples contained ∼3 times more embedded BC particles. The morphological configuration of embedded BC particles is closest to the core–shell structure commonly used in models and it is theoretically expected to show the largest absorption enhancement. In addition, the embedded particles for the high-*E*_abs-obs-TD_ samples had significantly larger area equivalent diameter than those for the low-*E*_abs-obs-TD_ samples (Fig. 3d) with the difference ranging between 58 and 159 nm at the 95% confidence level, suggesting that the BC particles associated with high-*E*_abs-obs-TD_ underwent substantial coating. Moreover, the thermodenuded particles during the high-*E*_abs-obs-TD_ period (Supplementary Table 3) showed a substantially larger fraction (36%) of thinly coated BC particles and a smaller fraction (3%) of embedded BC particles compared with the simultaneously collected ambient sample (Fig. 3b), consistent with the presence of semi-volatile components in the coatings on BC particles.

A similar analysis for particles collected at the urban T0 site (14 km northeast of the Sacramento downtown, CA, USA) during the Carbonaceous Aerosols and Radiative Effects Study (CARES) campaign is also shown in Supplementary Table 3 and the fractions of each type are shown in Fig. 3b. The embedded fraction was substantially higher at Detling (12%) than in Sacramento during the CARES campaign (1%), where only small *E*_abs_ is observed even at relatively large photochemical age (up to ∼15 h)^14^. In addition, the convexity^42^, which is associated with the compactness of the BC-containing particles, was higher for Detling (0.82±0.005) than CARES (0.76±0.005), suggesting different particle morphology at the two sites. This result indicates that mixing state may play an important role in determining *E*_abs_. However, comparable electron microscopy measurements are not available for the CalNex campaign^14^, where small *E*_abs_ values are observed even though similar SP-AMS measurements show substantially larger *R*_BC_ compared to the Detling measurements. Therefore, the hypothesis that the morphological details of the internal mixing-state significantly influence the absorption enhancement needs additional evidence with further field studies covering a range of different environments.

## Discussion

Our field results demonstrate clearly that coatings can substantially enhance light absorption by BC emitted from mixed fossil fuel and residential solid fuel combustion sources. Photochemical aging can play an important role in BC's absorption enhancement by coating BC particles with condensable materials. Comparison with results from other field campaigns indicates that the influence of coatings on BC absorption may be source and regionally specific. We hypothesize that mixed combustion sources and atmospheric transformations can produce favourable conditions of enhanced BC absorption by coatings, including BrC. Our analysis of absorption enhancement as a function of morphology, mixing state, coating thickness and the amount of low-volatility and semi-volatile BrC at wintertime Detling supports our hypothesis. We highlight that the effects of BrC, in particular the low-volatility BrC, on BC's absorption enhancement at short wavelengths should be considered in future studies. Our findings should be evaluated by future measurements of atmospherically processed BC particles from mixed combustion sources at other locations. We recommend that models treat the variability of BC's absorption to account for the dynamic aging processes and regional variations in sources by calibrating Mie models to measurements. Continued assessment of the absorption enhancement by BC coatings (including BrC) through comparing observations and observationally constrained calculations is necessary to develop robust numerical models, in order to accurately quantify the past, present and future contributions of absorbing particles to climate change.

## Methods

### Thermodenuder

A thermodenuder (TD, Aerodyne Research, Inc., Billerica, MA, USA) was installed upstream of all the instruments used in this study to evaporate semi-volatile particulate components. The flowrate through the TD was 2.3 l.p.m., resulting in a residence time of 5.3 s, which was shorter than the 8.5 s residence time in the study by Cappa *et al*.^14^

Ambient particles and heated particles were alternately sampled during 5–15 February 2012 with TD temperatures of 120 and 250 °C. Each ambient-TD cycle included 10-min ambient measurements and 10-min TD measurements. Unless stated otherwise, the *E*_abs_ values derived from 250 °C thermodenuding were reported.

### Photoacoustic soot spectrometer

A three-wavelength photoacoustic soot spectrometer (PASS-3, Droplet Measurement Technologies, Inc., Boulder, CO, USA) was used to continuously measure the absorption and scattering coefficients of fine particles at 405, 532 and 781 nm. In the PASS-3, the particle laden sample is illuminated with a modulated laser. The energy absorbed from the incident light is thermally transferred to the air surrounding the particle and the subsequent air expansion produces a sound wave^26^. The sound wave is measured by a microphone that was calibrated at 532 nm at Droplet Measurement Technologies, Inc. using NO_2_ (ref. 43). The microphone calibration is independent of wavelength and the lasers at 405, 532 and 781 nm were calibrated using a laser power meter. The calibration was checked using size selected fullerene soot particles (Alpha Aesar, Ward Hill, Massachusetts) following the procedures described by Flowers *et al*.^44^ An NO_2_ scrubber was installed upstream of the PASS-3 to remove the interference of NO_2_ absorption at 405 nm. Background measurements of filtered air were made every 2.5 min to allow for correction of changes in gas composition. During the Detling measurements, low 532 nm laser power and drifts caused large variations of the signal in this channel. Therefore, 
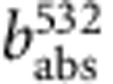
 was used as a qualitative measurement only and is not included here. Allan variance plots were made before the measurements to evaluate the precision and stability of the performance of the PASS-3 (refs 45, 46). The precision at 2.5 min integration time was 0.5 M m^−1^ for 
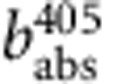
 and 0.3 M m^−1^ for 
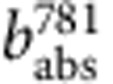
. Zero tests using high-efficiency particulate arrestance filters suggested that the gas-phase absorption was within the precision of the PASS-3 measurements. The upper limit of the gas-phase contribution to absorption was estimated to be 4 and 6% at 405 and 781 nm, respectively. The relative humidity inside the PASS-3 instrument was smaller than 30%, suggesting that the particles were dry by the time they were measured. The low relative humidity inside the PASS-3 instrument was due to warm up of the cold air brought into the warmer container and the relatively high temperature (20–30 °C) in the PASS-3 measurement cell induced by the electronics.

To verify the PASS-3 measurements, the absorption measured by the PASS-3 was compared with the absorption calculated from a 7-wavelength Aethalometer (MAGEE Scientific, model AE31) that operated simultaneously at the same site^47^. The PASS-3 measured absorption correlated well with the Aethalometer-derived absorption with Pearson's *r*≥0.94 at 405 and 781 nm. Major axis line-fitting suggests that the PASS-3 and Aethalometer measurements agreed well, with slopes close to 1 (0.934±0.004 at 405 and 0.918±0.004 at 781 nm) and intercepts slightly negative (−2.724±0.085 and −1.847±0.037 M m^−1^).

### SP2

The refractory BC (rBC) mass concentration was quantified using an SP2 (Droplet Measurement Technologies, Inc., Boulder, CO, USA). Measurement principles of the SP2 have been described in detail in previous publications^27^,^28^. In brief, the SP2 selectively measures the rBC-containing particles using a diode-pumped 1064, nm Nd:YAG laser on a single-particle basis. The rBC-containing particles absorb the energy emitted by the laser and are heated to their vaporization temperature and incandesce. The peak intensity of the incandescence signal is proportional to the mass of rBC, allowing for the quantification of rBC mass concentration. The SP2 measures single rBC-containing particles with an rBC mass range of ∼3–300 fg (ref. 27). The size of the rBC was calculated assuming a density of 1.8 g cm^−3^ and assuming spherical geometry with no internal voids^48^. Size-selected fullerene soot (Alfa Aesar, Ward Hill, Massachusetts; Stock# 40971, Lot# L18U002) was used for the SP2 mass calibration^49^. Contributions of rBC particles outside the detection window—which can vary with rBC source—were quantitatively estimated by fitting time-dependent rBC size distributions assuming a bimodal lognormal shape. The good correlation between absorption and [rBC] (Supplementary Fig. 4) suggests that the lognormal correction was sufficient to account for the rBC mass outside the SP2 detection limit.

### HR-ToF-AMS

An Aerodyne HR-ToF-AMS (Aerodyne, Research Inc., Billerica, MA, USA) was deployed to measure the non-refractory organics, sulfate, nitrate, ammonium and chloride in submicron particles with 2-min time resolution. The HR-ToF-AMS configuration and principle of operation have been described in detail previously^29^,^30^. Briefly, particles that are focused and accelerated by an aerodynamic lens impact a heated vaporizer (600 °C). The non-refractory components in the particles are flash vapourized and ionized, and the ionized fragments are measured by a high-resolution time-of-flight mass spectrometer.

### SP-AMS

The SP-AMS (Aerodyne Research, Inc., Billerica, MA, USA) incorporates a 1064, nm Nd:YAG laser vaporizer (the same laser as used in the SP2 instrument) into the standard Aerodyne HR-ToF-AMS to detect rBC via laser-induced heating and vaporization^31^. During 5–15 February 2012, the tungsten thermal vaporizer was removed from the SP-AMS so that only rBC and the rBC-associated non-refractory particulate matter (including organics, sulfate, nitrate, ammonium and chloride) were detected. By comparing the ambient and thermodenuded OM that was associated with BC (normalized to [rBC] to account for particle losses in the heated TD), we find that on average 24% of OM that was internally mixed with rBC remained associated with the rBC particles after heating to 250 °C. This result is consistent with a previous study^50^ that shows large contribution (∼11%) of organic components to particulate residuals after particle heating at 300 °C.

### Electron microscopy analysis

Single particles were collected on Nuclepore filters (100 nm pores) using an aspiration sampling technique. The sample collection time was 3–6 h depending on the particle concentration. For each collection time period, two filter samples, one representing ambient and the other representing heated (at 120 °C through a TD) particles, were collected simultaneously. This TD was different from the TD used for the PASS-3, HR-ToF-AMS and SP-AMS, and therefore it may remove a different amount of material. After collection, the samples were stored in the dark before analysis. The particle images were acquired using a field emission scanning electron microscope (Hitachi S-4700). We note that only particles larger than 100 nm were analysed to reduce uncertainties in particle classification. In addition, the number of embedded BC-containing particles was likely underestimated because (1) electron microscopy images only the surface features of the particles, thereby the BC-containing particles that were entirely engulfed by other materials and completely lost their original BC morphology could not be identified and (2) the electron microscopy analysis was conducted under vacuum, so evaporation of semi-volatile coatings on BC particles could not be avoided. Therefore, the number fraction for each particle type derived from the electron microscopy analysis was semi-quantitative.

### Calculation of *E*_abs-obs-TD_

The TD method is described by the following equation


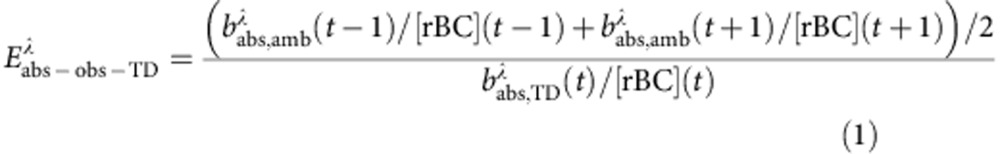


where 
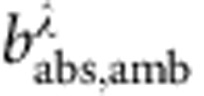
 and 
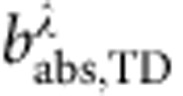
 represent the absorption coefficients of ambient and thermodenuded particles, respectively; [rBC] is the mass concentration of rBC measured by the SP2; *t* represents the TD measurement, and *t*−1 and *t*+1 represent the times corresponding to ambient measurements before and after the TD measurement, respectively. Over 95% of the *E*_abs-obs-TD_ values were >1, with the values smaller than 1 resulting from temporal atmospheric variability during the sequential ambient and TD measurements.

### Calculation of *E*_abs-obs-MAC_

In the calculation of *E*_abs-obs-MAC_, the measurements were binned by the *R*_BC_ intervals, and the *MAC* of BC was determined as the slope of the line-fitting of 
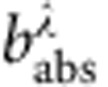
 as a function of [rBC] for each measurement section (Supplementary Fig. 4). We examined the linear regression, major axis (also called orthogonal regression), and standardized major axis (also called reduced major axis) least square line-fitting methods. The linear regression method only accounts for the errors in the *Y* direction, while the other two methods take into account errors in both *X* and *Y* directions. The standardized major axis is the major axis calculated on standardized data. The standardized major axis is typically used when *X* and *Y* data are not measured on comparable scales^51^, which is appropriate for the *b*_abs_ versus [rBC] line-fitting in this study. In addition, comparison of the three fitting methods results showed that the slopes derived from standardized major axis were in between those derived from the linear regression and major axis fitting methods (Supplementary Fig. 2). An additional method was to take the average point-to-point *b*_abs_/[rBC] as the *MAC* value for each *R*_BC_ interval, defined as the ‘ratio method.' The ratio method did not give the same results as those derived from the slope methods. We suspect that this difference was due to a systematic offset in the PASS-3 measurements that affected the ratio method but not the slope methods. For these reasons, we used the slopes derived from the standardized major axis fitting as the *MAC* values in this study. *E*_abs-obs-MAC_ was calculated as the ratio of the calculated *MAC* to the reference *MAC* of uncoated BC.

### Apportionment of the absorption at 405 nm

The measured 
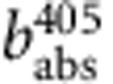
 was the sum of the absorption of BC, BrC (both internally and externally mixed with BC), and the enhanced absorption due to the lensing effect of coatings on BC. Upon heating at 250 °C, the semi-volatile OM and semi-volatile inorganic species evaporated, leaving only BC and low-volatility non-BC materials. Unless stated otherwise, the temperature threshold for separating semi-volatile and low-volatility components was defined as 250 °C in this study. 
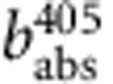
 can be apportioned using 
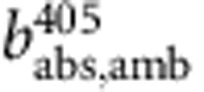
 and 
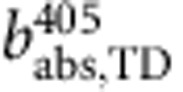
 (at 250 °C) based on the following assumptions: (1) BrC absorbs at 405 nm but not at 781 nm (ref. 24); (2) the absorption of the residual BC particles (after heating) follows the inverse wavelength dependence; and (3) to a first-order approximation the lensing-driven enhancement for BC particles is wavelength independent, that is, 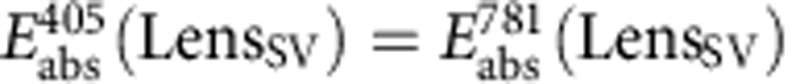 for the same BC-containing particle^52^. Note that the measured ambient 
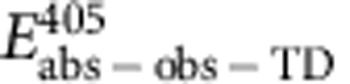
 included the lensing-driven enhancement and the enhancement induced by evaporation of BrC_SV_, and further 
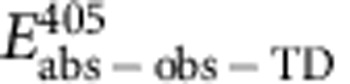
 was affected by BrC_LV_ as discussed in the main text. Therefore 
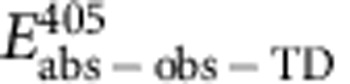
 is different from 
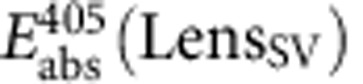
 used in the equations below: 
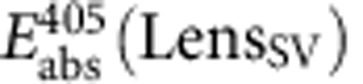
 excludes the effects of BrC (BrC_LV_ and BrC_SV_) and represents the lensing-driven enhancement only. In comparison with 
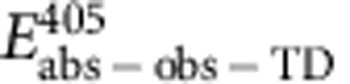
, the measured 
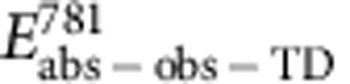
 was due to the lensing-driven enhancement only, that is, 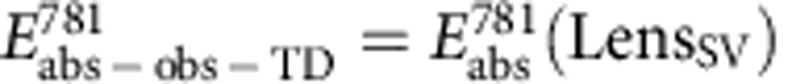. The apportionment is described by the following equations with the variables described in Supplementary Table 1:


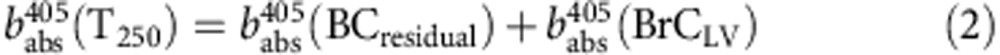



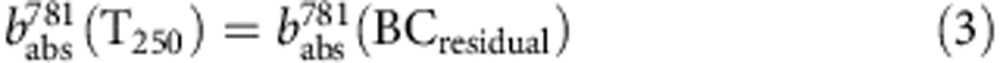



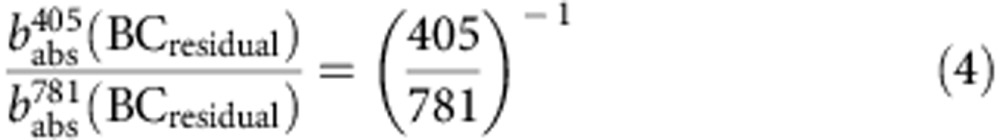



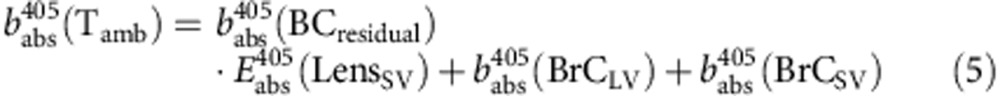



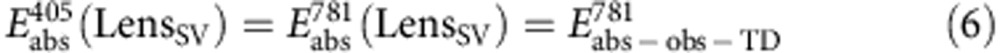


From equations (2, 3, 4), 
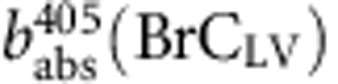
 and 
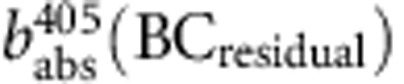
 were solved; 
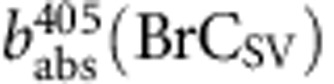
 was then derived using equations (5) and (6).

Similar apportionment of 
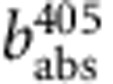
 was performed using the 
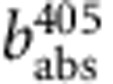
 for particles that were thermodenuded at 120 °C. In this case, the BrC_LV_ was referred to as the fraction of BrC that remained in the particles after thermodenuding at 120 °C. The apportioned 
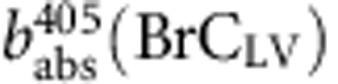
 at 120 °C and 250 °C correlated in time with a Pearson's *r* of 0.8, and the latter was 19% lower on average, which was likely because of the absorption of BrC that evaporated between 120 and 250 °C. Since charring of OM is unlikely to take place at 120 °C, the correlation of apportioned 
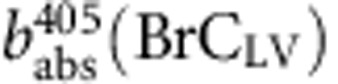
 at 120 and 250 °C suggests that 
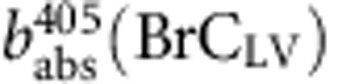
 at 250 °C was a real signal rather than an artefact resulting from pyrolyzed OM. This result is consistent with previous studies that confirm that the interference of pyrolyzed OM on the absorption measurements is <1.7% at 300 °C (ref. 53).

### Non-fossil fractions of oxygenated organic carbon

Radiocarbon (^14^C) analysis was used to distinguish the fossil and non-fossil carbon fractions of the oxygenated organic aerosols retrieved from the HR-ToF-AMS analysis. Non-fossil carbon fraction (*f*_NF_) was calculated as the ratio of ^14^C/^12^C for the sample to ^14^C/^12^C in 1950 after correcting for field blanks and nuclear weapon tests in the 1950s and 1960s (ref. 54). The *f*_NF_ for total carbon (*f*_NF,TC_) in Detling has been reported by Crilley *et al*.^55^, in which the total carbon is the sum of organic carbon and elemental carbon measured with a Sunset thermal-optical carbon analyser. *f*_NF_ of BC (*f*_NF,BC_) in Detling was assumed to be the wood burning fraction of BC, apportioned using the Aethalometer measurements and a source apportionment model^56^ as reported in Crilley *et al*. The average *f*_NF,TC_ and *f*_NF,BC_ at Detling during ClearfLo are 64±7% and 30±13% (ref. 55), respectively.

Here, we expanded the Crilley *et al*. analysis to analyse the *f*_NF_ for oxygenated organic aerosols using the method described in Zotter *et al*.^54^ The equation we used for the *f*_NF_ calculation is as follows


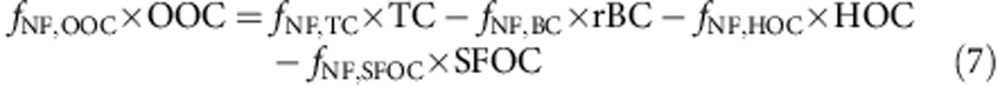


where HOC, SFOC and OOC are the organic carbon concentrations for hydrocarbon-like organic aerosol, solid fuel organic aerosol, and oxygenated organic aerosol factors, respectively. The organic carbon concentration of the factor was converted from the corresponding factor OM concentration, using the OM to organic carbon ratios derived from the factor spectra. rBC is the ambient refractory BC concentration measured by the SP2. TC is the total carbon concentration, equivalent to the sum of HOC, SFOC, OOC, and BC concentrations. *f*_NF,TC_ and *f*_NF,BC_ values are the same as reported in Crilley *et al*.^55^ We used the *f*_NF,TC_ and *f*_NF,BC_ values for 5–12 February 2012 that overlapped the *E*_abs_ measurements. *f*_NF,HOC_ and *f*_NF,SFOC_ are the non-fossil carbon fractions for HOC and SOFC, respectively. Since *f*_NF,HOC_ and *f*_NF,SFOC_ were not explicitly known, we performed sensitivity analysis using a pseudo Monte Carlo method. In this method, *f*_NF,HOC_ was varied between 0 and 0.3, representing its substantial fossil emissions and accounting for the potential contribution of non-fossil cooking and biofuel combustion (for example, biodiesel) emissions that were not separated from HOC; *f*_NF,SFOC_ was varied between 0.9 and 1.0 to consider the dominance of wood burning and account for the contribution of residential (fossil) coal burning to SFOC.

The apportionment result (Supplementary Fig. 5) showed that the *f*_NF,OOC_ ranged from 0.75 to 0.93, suggesting that the majority of oxygenated organic aerosols was from non-fossil sources in Detling during ClearfLo.

### Mie and RDG model calculations

The Mie calculations were performed using the same Igor code (Wavemetrics Inc) as in Cappa *et al*.^14^, which was adapted from the Fortran code of Bohren and Huffman^57^. The input size distributions for the calculation were constrained by observations using the same set of measurements (from the same types of instruments) and methods as detailed in Cappa *et al*., that is, the core BC size distribution was constrained by the SP2 measurements, the coated BC-containing particle size distribution was determined by *R*_BC_ and the core BC size distribution assuming that the coated components were evenly distributed around BC particles, and the non-BC particle size distribution was derived by subtracting the SP2-measured BC size distribution from the total particle size distribution measured by a scanning mobility particle sizer.

In the RDG calculation, the number of spherules per particle was calculated by the assumed spherule size and the SP2-measured spherical equivalent volumes of the overall BC core. It was assumed that the amount of coating on each spherule was proportionally the same as for the total particle, that is, that *R*_BC_ was the same for the spherule as for the total particle. This assumption is equivalent to having the ratio between the coated diameter and the core diameter conserved between the standard Mie (total particle) formulation and the RDG formulation. The absorption enhancements for the RDG cases were calculated analogously to the Mie case.

The refractive index of BC was assumed to be *n*=1.88+0.8*i* (ref. 14). Since the imaginary refractive index of BrC (*k*_BrC_) is highly uncertain and can vary greatly, we performed calculations to estimate the *k*_BrC_ that resulted in good agreement between predicted and measured *E*_abs-MAC_ and *E*_abs-TD_ at 405 nm. It was assumed that the refractive index was a bulk average for OM.

## Additional information

**How to cite this article:** Liu, S. *et al*. Enhanced light absorption by mixed source black and brown carbon particles in UK winter. *Nat. Commun.* 6:8435 doi: 10.1038/ncomms9435 (2015).

## Supplementary Material

Supplementary Information Supplementary Figures 1-5, Supplementary Tables 1-3 and Supplementary Reference
