# Supplementary material for: Enhanced light absorption by mixed source black and brown carbon particles in UK winter
Source: Nat Commun. 2015 Sep 30;6:8435. doi: 10.1038/ncomms9435 (PMC4598716; doi:10.1038/ncomms9435)
Supplement: Supplementary Information — Supplementary Figures 1-5, Supplementary Tables 1-3 and Supplementary Reference [file ncomms9435-s1.pdf]

Supplementary Figures

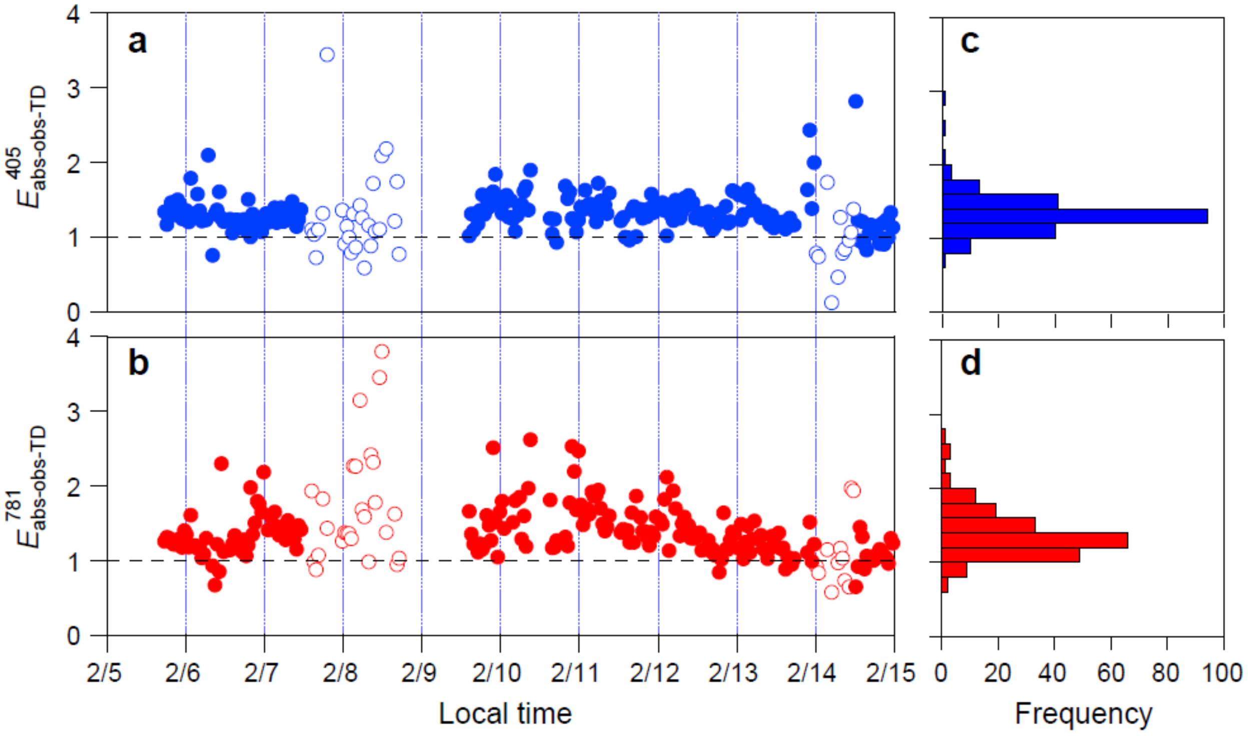

Supplementary Figure 1 | Time series and frequency distribution of $E_{abs-obs-TD}$. **(a, b)** show time series of $E_{abs-obs-TD}$ at 405 nm and 781 nm, respectively. The solid points represent the $E_{abs-obs-TD}$ values used for the analysis. The open circles represent the $E_{abs-obs-TD}$ values derived during two clean (low signal) periods, which were excluded for data analysis to ensure data quality. Including the low-signal $E_{abs-obs-TD}$ values resulted in nearly the same trends and figures as those without them (the changes of the R_{BC}-binned averages were smaller than 2%). **(c, d)** show frequency distribution of $E_{abs-obs-TD}$ at 405 nm and 781 nm, respectively.

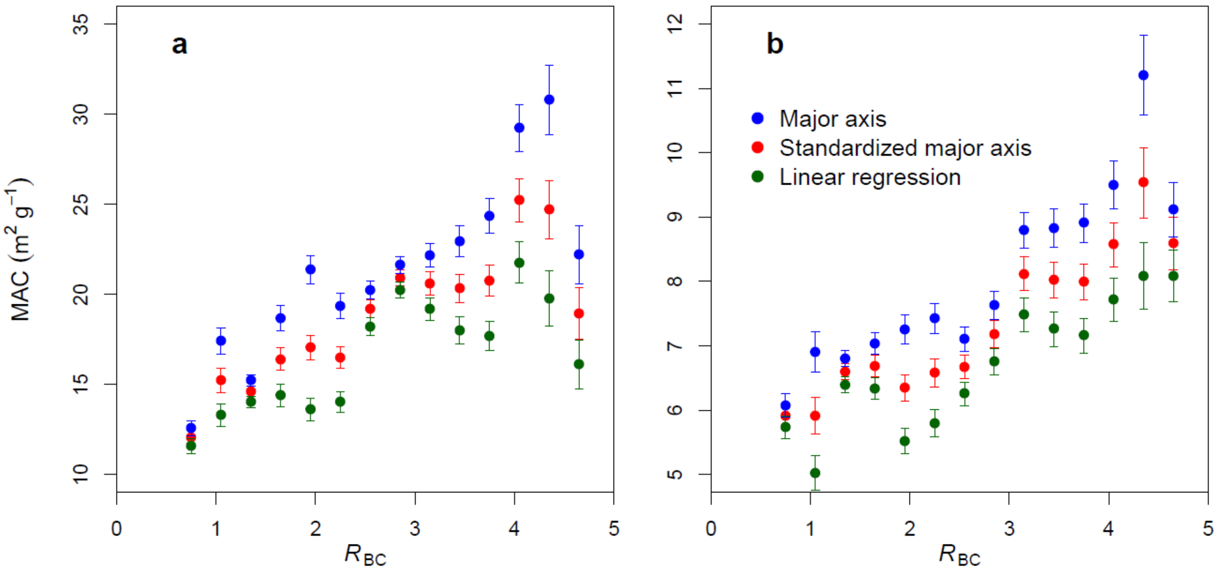

Supplementary Figure 2 | Comparison of the three line-fitting methods. Comparison of the absolute *MAC* values at (**a**) 405 nm and (**b**) 781 nm derived from major axis (blue), standardized major axis (red), and linear regression (green) fitting methods. Each point represents the slope of the best fit for each R_{BC} interval. The standardized major axis results are presented in Fig. 1 in the main text.

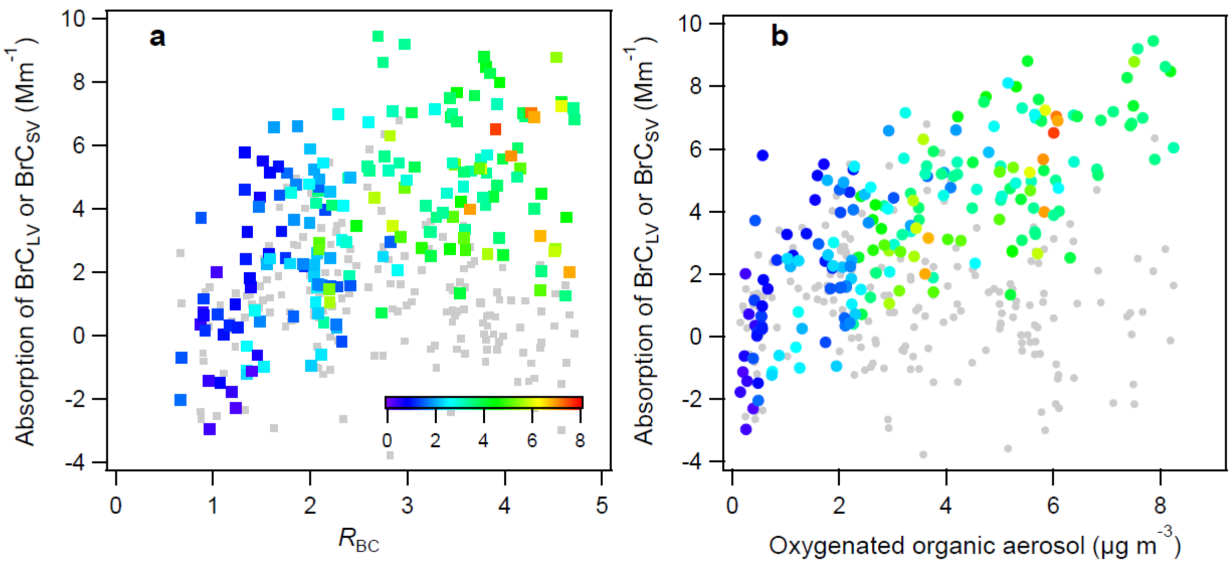

Supplementary Figure 3 | Absorption of BrC$_{LV}$ and BrC$_{SV}$ versus R_{BC} and oxygenated organic aerosol. Apportioned $b_{abs}^{405}(BrC_{LV})$ (colored points) and $b_{abs}^{405}(BrC_{SV})$ (grey dots) versus (**a**) R_{BC} and (**b**) the oxygenated organic aerosol factor concentration derived from the HR-ToF-AMS measurements. The $b_{abs}^{405}(BrC_{LV})$ points are color-coded by sulfate concentration (μg m^{-3}) measured by the HR-ToF-AMS, with the color scale shown in (**a**). $b_{abs}^{405}(BrC_{LV})$ correlated to R_{BC} and the oxygenated organic aerosol factor concentration with Pearson's r of 0.6 and 0.7, respectively. The negative values were due to the atmospheric variability in the sequential ambient and TD measurements that were used for the absorption apportionment.

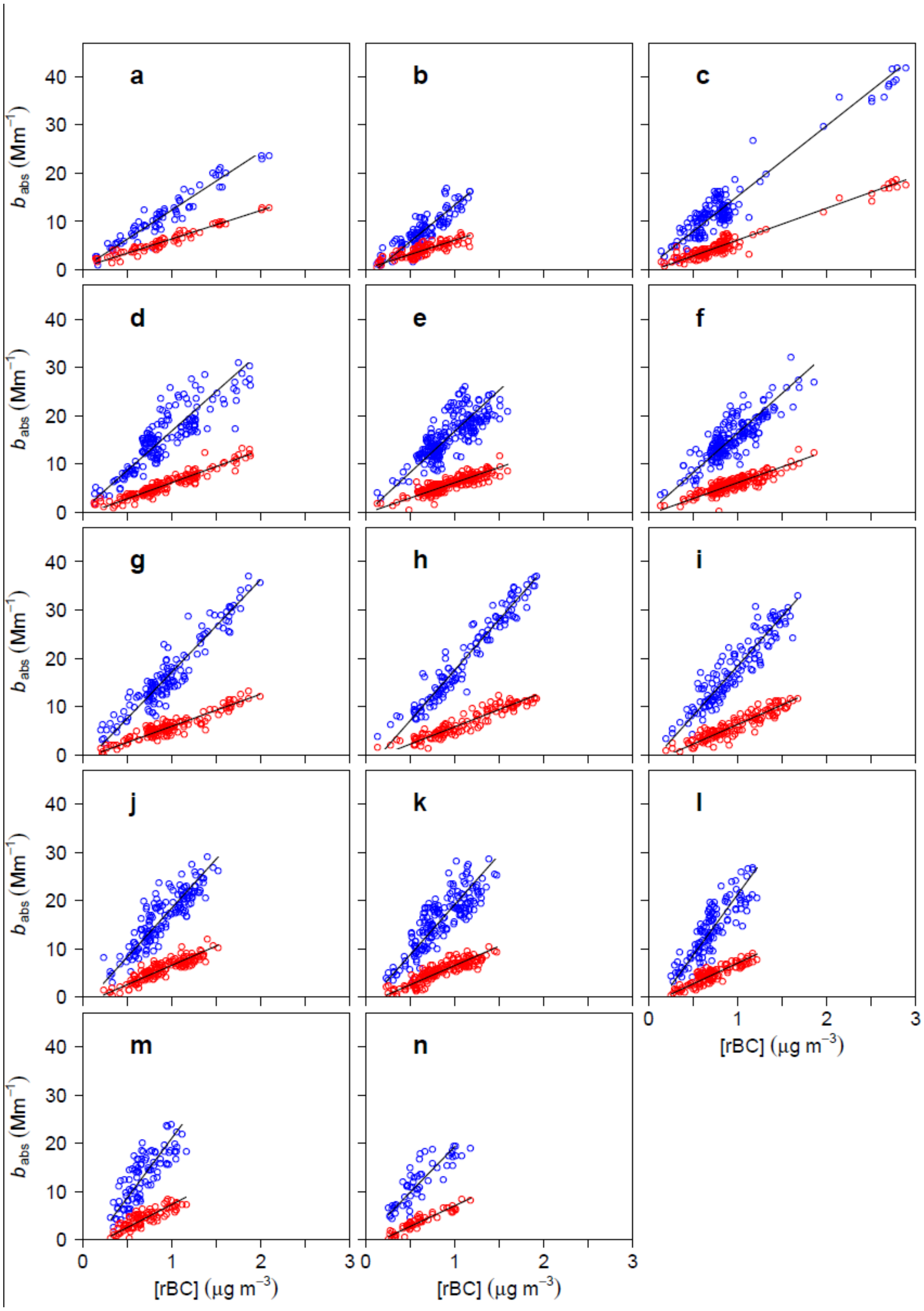

Supplementary Figure 4 | b_{abs} versus rBC concentration. b_{abs} as a function of [rBC] at 405 nm (blue) and 781 nm (red), for R_{BC} intervals of (0.6, 0.9], (0.9, 1.2], (1.2, 1.5], (1.5, 1.8], (1.8, 2.1], (2.1, 2.4], (2.4, 2.7], (2.7, 3.0], (3.0, 3.3], (3.3, 3.6], (3.6, 3.9], (3.9, 4.2], (4.2, 4.5], (4.5, 4.8] in (**a-n**), respectively. (The round brackets indicate greater than, and the square brackets indicate

equal to or smaller than.) The lines represent the best fits using the standardized major axis fitting method.

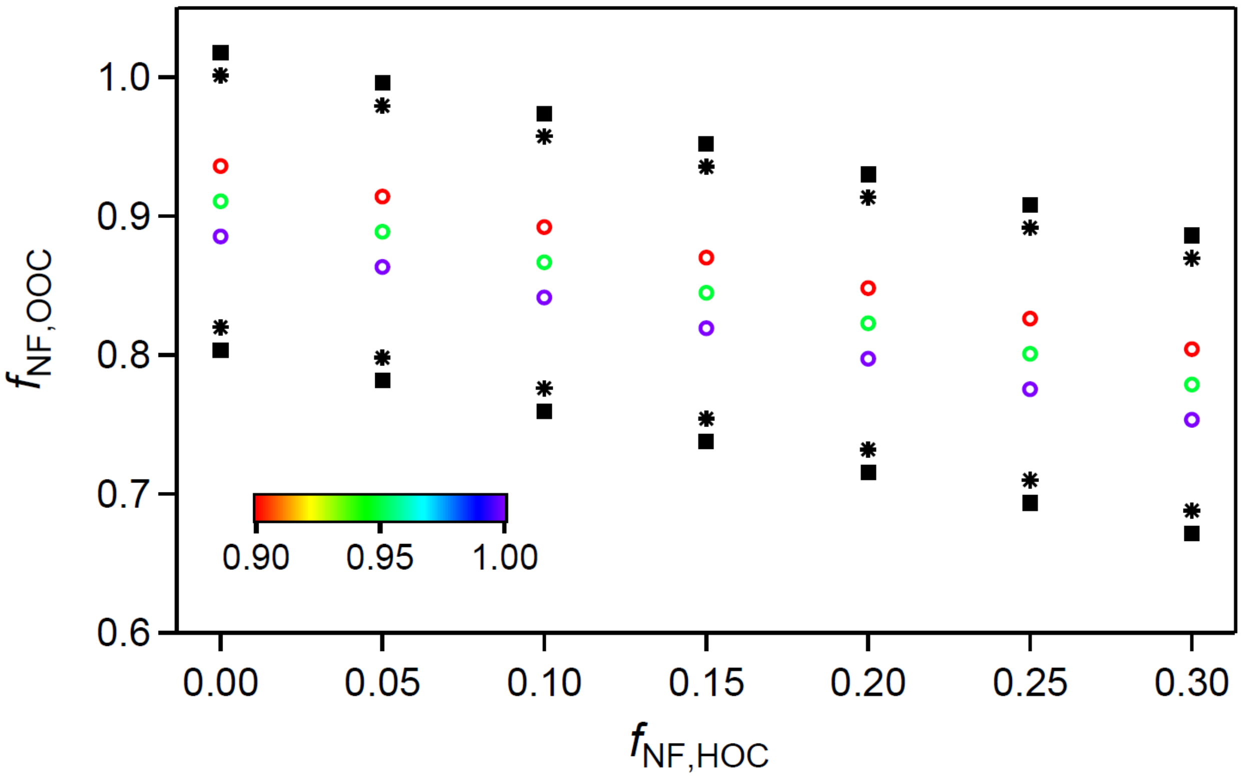

Supplementary Figure 5 | Variation of $f_{NF,OOC}$ with $f_{NF,HOC}$ and $f_{NF,SFOC}$. $f_{NF,OOC}$ was calculated using a range of $f_{NF,HOC}$ (0–0.3) and $f_{NF,SFOC}$ (0.9–1.0) values, with the results shown in colored circles. The colors of the circles indicate $f_{NF,SFOC}$ as shown in the color bar. The stars represent uncertainty bounds derived from the uncertainty of $f_{NF,BC}$, and the squares represent uncertainty bounds derived from the uncertainty of $f_{NF,TC}$.

Supplementary Tables

Supplementary Table 1 | Description of acronyms used for instruments and variables in the main text and variables used for equations (2)–(6) in Methods.

Acronym	Description	Acronym	Description
**Acronyms for instruments**			
TD	Thermodenuder	SP-AMS	Soot particle aerosol mass spectrometer
SP2	Single particle soot photometer	HR-ToF-AMS	High resolution time-of-flight aerosol mass spectrometer
**Acronyms for variables**			
BC	Black carbon (general description)	rBC	Refractory black carbon (specific to measurements)
MAC	Mass absorption cross section	BrC	Brown carbon
BrC_{LV}	Low-volatility brown carbon	BrC_{SV}	Semi-volatile brown carbon
b_{abs}	Absorption coefficient	E_{abs}	Absorption enhancement of black carbon
$E_{abs\text{-}obs\text{-}TD}$	Observed absorption enhancement by the TD method	$E_{abs\text{-}obs\text{-}MAC}$	Observed absorption enhancement by the MAC method
$E_{abs\text{-}calc\text{-}TD}$	Calculated absorption enhancement relative to thermodenuded residuals	$E_{abs\text{-}calc\text{-}MAC}$	Calculated absorption enhancement relative to uncoated black carbon
OM	Organic mass	R_{BC}	Mass ratio of non-refractory black carbon-associated materials to black carbon
k_{BrC}	Imaginary refractive index of brown carbon	$k_{BrC,amb}$	Imaginary refractive index of ambient brown carbon
$k_{BrC,TD}$	Imaginary refractive index of thermodenuded brown carbon	RDG	Rayleigh-Debye-Gans approximation
**Acronyms used in Equations (2)–(6)**			
$b_{abs}^{405}(T_{amb})$	Measured b_{abs}^{405} for ambient particles	$b_{abs}^{405}(T_{250})$	Measured b_{abs}^{405} for thermodenuded (250 °C) particles
$b_{abs}^{781}(T_{250})$	Measured b_{abs}^{781} for thermodenuded (250 °C) particles	$b_{abs}^{405}(BC_{residual})$	b_{abs}^{405} of residual BC particles after thermodenuding (250 °C)
$b_{abs}^{781}(BC_{residual})$	b_{abs}^{781} of residual BC particles after thermodenuding (250 °C)	$b_{abs}^{405}(BrC_{LV})$	b_{abs}^{405} of low-volatility BrC
$b_{abs}^{405}(BrC_{SV})$	b_{abs}^{405} of semi-volatile BrC	$E_{abs}^{405}(Lens_{SV})$	Lensing-driven absorption enhancement due to semi-volatile materials at 405 nm
$E_{abs}^{781}(Lens_{SV})$	Lensing-driven absorption enhancement due to semi-volatile materials at 781 nm		

Supplementary Table 2 | Campaign-average apportioned b_{abs}^{405} in this study and in Lack et al.[1]

	[1]BC (%)	BrC_{LV} (%)	BrC_{SV} (%)	Total BrC (%)	Lensing-driven absorption (%)
Detling	53	24	7	31	16
Lack et al.	54	-	-	27	19

[1]The absorption of BC derived here was the absorption of residual BC-containing particles after heating at 250 °C.

Supplementary Table 3 | Statistics of BC-containing particles.

Project	Sample ID	Particle number	Collection time start (UTC)	Collection time end (UTC)	Number fraction (%)			
					Embedded	Partly coated	Thinly coated	Partially-encapsulated and/or surface attached
ClearfLo	#1A	858	2/10/2012 19:58	2/11/2012 2:08	14	67	13	6
	#2A	1329	2/11/2012 8:59	2/11/2012 15:30	18	55	12	15
	#2D	500	2/11/2012 9:09	2/11/2012 15:40	3	51	36	10
	#3A	1306	2/5/2012 19:39	2/5/2012 23:49	6	63	11	20
	#4A	711	2/9/2012 15:16	2/9/2012 18:06	7	71	14	8
CARES	#2A	563	6/25/2010 9:01	6/25/2010 11:01	1	79	20	0
	#3A	471	6/25/2010 16:31	6/25/2010 18:31	1	84	9	6

Summary of BC-containing particle classification for the ClearfLo (at Detling) and CARES (at Sacramento) projects as estimated from the electron microscopy analysis. The suffix in sample ID represents ambient (A) and thermodenuded (D) samples. For the Detling samples, #1 and #2 were associated with high $E_{\text{abs-obs-TD}}$ values, and # 3 and #4 were associated with low $E_{\text{abs-obs-TD}}$ values.

Supplementary Reference

1 Lack, D. A. *et al.* Brown carbon and internal mixing in biomass burning particles. *Proc. Natl Acad. Sci. USA* **109**, 14802-14807 (2012).
